# Copper Binding Features of Tropomyosin-Receptor-Kinase-A Fragment: Clue for Neurotrophic Factors and Metals Link

**DOI:** 10.3390/ijms19082374

**Published:** 2018-08-12

**Authors:** Antonio Magrì, Diego La Mendola

**Affiliations:** 1 Institute of Biostructures and Bioimages, National Council of Research (CNR), Via Paolo Gaifami 18, 95126 Catania, Italy; leotony@unict.it; 2 Consorzio Interuniversitario di Ricerca in Chimica dei Metalli nei Sistemi Biologici (CIRCMSB), via Celso Ulpiani, 27, 70125 Bari, Italy; 3 Department of Pharmacy, University of Pisa, Via Bonanno Pisano 6, 56126 Pisa, Italy

**Keywords:** neurotrophin, copper, metal complexes, peptide, receptor

## Abstract

The nerve growth factor (NGF) is a neurotrophin essential for the development and maintenance of neurons, whose activity is influenced by copper ions. The NGF protein exerts its action by binding to its specific receptor, TrkA. In this study, a specific domain of the TrkA receptor, region 58–64, was synthesized and its copper(II) complexes characterized by means of potentiometric and spectroscopic studies. The two vicinal histidine residues provide excellent metal anchoring sites and, at physiological pH, a complex with the involvement of the peptide backbone amide nitrogen is the predominant species. The TrkA peptide is competitive for metal binding with analogous peptides due to the N-terminal domain of NGF. These data provide cues for future exploration of the effect of metal ions on the activity of the NGF and its specific cellular receptor.

## 1. Introduction

The neurotrophins are a family of growth factors essential for neural survival, development, function, and plasticity [1,2,3]. They include nerve growth factor (NGF), brain-derived neurotrophic factor (BDNF), NT-3 (neurotrophin 3), and NT-4 (neurotrophin 4) [4]. Neurotrophins exert their biological functions by interacting with two cellular receptors: tyrosine receptors kinases (TrkA, TrkB, and TrkC) specific for each protein, and the common neurotrophin receptor P75^NTR^, a member of the tumor necrosis factor receptor superfamily [5,6].

**Scheme 1:**
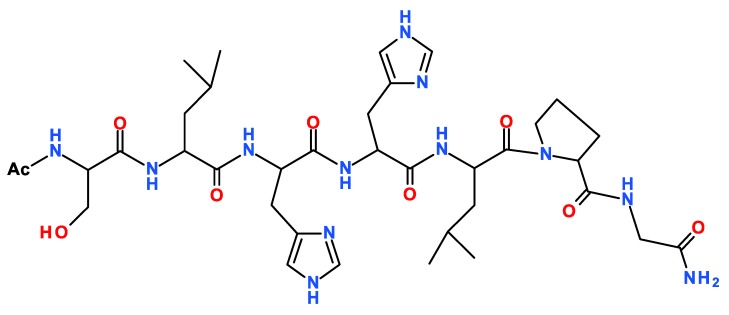
Schematic view of the primary sequence of TrkA(58–64) and Ac-SLHHLPG-NH_2_ peptide.

The first to be discovered and the best-characterized neurotrophin is NGF [7]. NGF, through binding to its specific receptor TrkA, promotes the development and maintenance of basal forebrain cholinergic neurons [8]. Metabolic dysfunction of NGF is involved in Alzheimer’s Diseases (AD) occurrence [9,10,11]. Metal ions play a role in AD [12,13,14] and copper and zinc ions can modulate NGF activity in the same brain areas affected by metal dyshomeostasis in pathological conditions [15,16,17]. Metal binding can induce NGF conformational changes that hinder the recognition process with its cellular receptors [18,19,20]. The N-terminal domain of NGF is involved in the TrkA recognition process, and peptides encompassing the first 14 residues are able to bind copper and zinc ions and to mimic whole protein activity in the presence of metal [21,22]. Zinc and copper levels are high in the synaptic cleft and extracellular metal ions also activate the Trk receptor [23,24,25,26]. Therefore, metal ions dyshomeostasis may interfere in the interaction between NGF and its receptor, so identifying possible binding sites of this metal ions in the NGF/TrKA complex is of interest. Recently, we demonstrated that homodimer NGF protein interacts in a “crab” shape with two TrkA units, and in particular with TrkA N-terminal region encompassing His-60 and His-61 [27]. This domain is an effective binding site for zinc(II) ions, suggesting that metal ions may interact directly with the NGF receptor influencing protein recognition processes. In this work, we report the copper(II) complex formation with the Ac-SLHHLPG-NH_2_ peptide encompassing the sequence 58–64 of the TrkA protein (Scheme 1). Two adjacent histidine residues represent the main copper binding site in different proteins and biological peptides as Aβ amyloid [28,29,30,31]. In the NGF-receptor, two TrkA units have these domains close each other to form a sort of pocket with four histidine imidazole nitrogen facing inward [27]. The ability of Ac-SLHHLG-NH_2_ peptide to coordinate copper(II) ions was investigated by means of potentiometry and spectroscopic techniques, including ultraviolet-visible (UV-vis), circular dichroism (CD), electron paramagnetic resistance (EPR), exploring 1:1 and 1:2 metal-to-ligand molar ratios, to mimic the interaction of metal with one and two TrkA units, respectively. Comparison with the stability constant values of analogous copper(II) complexes, formed with a peptide encompassing the NGF N-terminal domain, indicated that the TrkA scaffold can compete with protein for metal ion binding.

## 2. Results

The protonation constants were previously determined (logβ_012_ = 12.80, logβ_011_ = 6.91) [27]. The measurements were carried out to verify peptide purity, as it was synthesized with a different approach by using microwave assisted peptide synthesis. The values obtained were the same as those reported within the range of experimental error [27].

**Figure 1:**
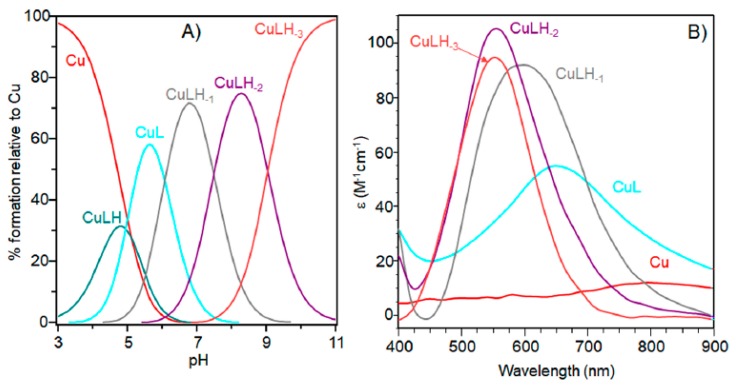
(**A**) Species distribution diagram for Cu-Ac-SLHHLPG-NH_2_ complexes at 1:1 copper-to-ligand molar ratio; and (**B**) ultraviolet-visible (UV-vis) spectra calculated using the computational program HYPERQUAD. [L] = 1 × 10^−3^ M. For clarity, the charges on the speciation plots were omitted.

The characterization of copper(II) complexes with Ac-SLHHLPG-NH_2_ showed the formation of only mononuclear 1:1 and1:2 bis-complexes. At a 1:1 molar ratio, the peptide started to bind metal ions above pH = 3.0 with the formation of the [CuLH]^3+^ species (Table 1 and Figure 1).

The logK value (logK = logβ_111_ − logβ_011_ = 3.70) (Table 1) was higher than that generally reported for the binding of an imidazole group without the involvement of other donor atoms different from water molecules. This value suggests that a carbonyl oxygen donor atom may be involved in copper binding, as found for analogous copper species formed with other linear peptides [32].

Increasing the pH, [CuL]^2+^ species were formed. The logβ value of 6.19 indicated a copper coordination environment involving both imidazole nitrogen atoms with subsequent formation of a 10-membered macrochelate [33,34,35]. Also for this complex species, the logβ was higher than those reported for analogous complex species formed by multihistidine-containing peptides, suggesting that the carbonyl group is still involved in metal binding [36].

**Figure 2:**
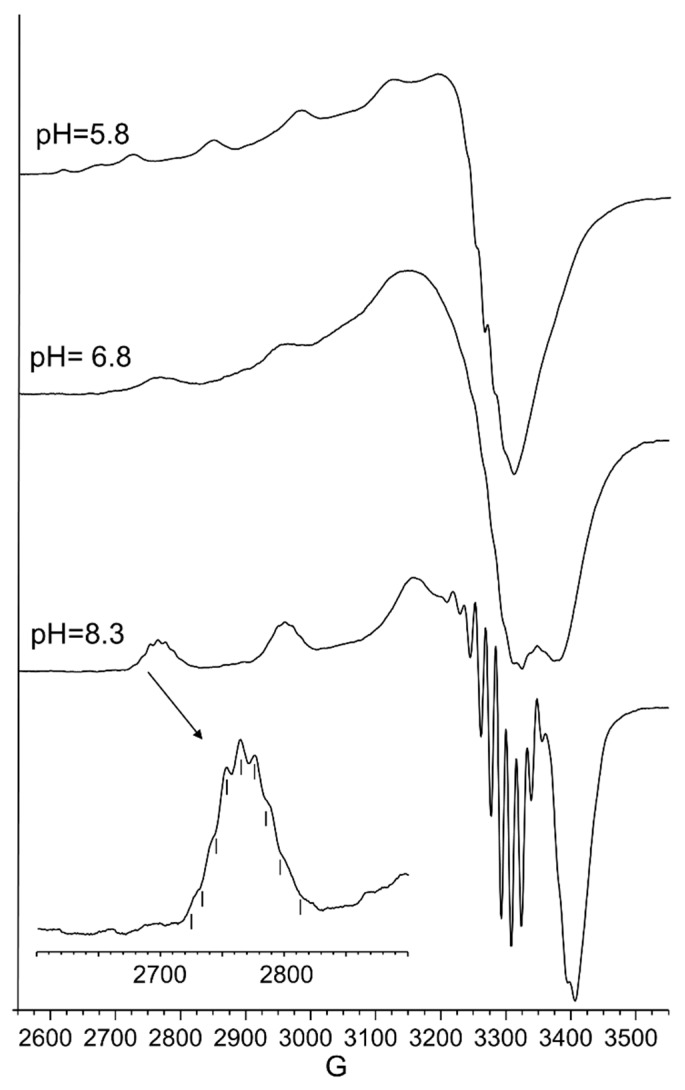
Aqueous frozen solution electron paramagnetic resistance (EPR) spectra at 150 K at different pH values of the Cu^2+^ -Ac-SLHHLG-NH_2_ system at 1:1 metal-to-ligand molar ratio ([Cu^2+^] = [L] = 1 × 10^−3^ mol dm^−3^). The magnification of the first band of EPR spectrum at pH 8.3 (black arrow) shows superhyperfine pattern superimposed on the first copper feature.

The UV-vis and EPR parameters (Table 2) support macrochelate formation with the presence of a Cu(2N_Im_, O_carbonyl_, O_water_) chromophore, as observed in similar peptides encompassing two vicinal histidine residues [35,37]. The percentage formation of [CuL]^2+^ was a maximum at pH 5.8. EPR spectrum carried out at this pH value (Figure 2) permitted us to obtain *g* and the hyperfine coupling constant assigned to this species. The hyperfine coupling constant (A_║║_) was lower than that reported for analogous complexes formed with peptides having two or more distal histidine residues, where A_║║_ in a range of 155–170 × 10^−4^ cm^−1^, confirming that the carbonyl oxygen is still involved in metal coordination environment so to determine a slight distortion of two imidazole histidine planar disposition.

**Figure 3:**
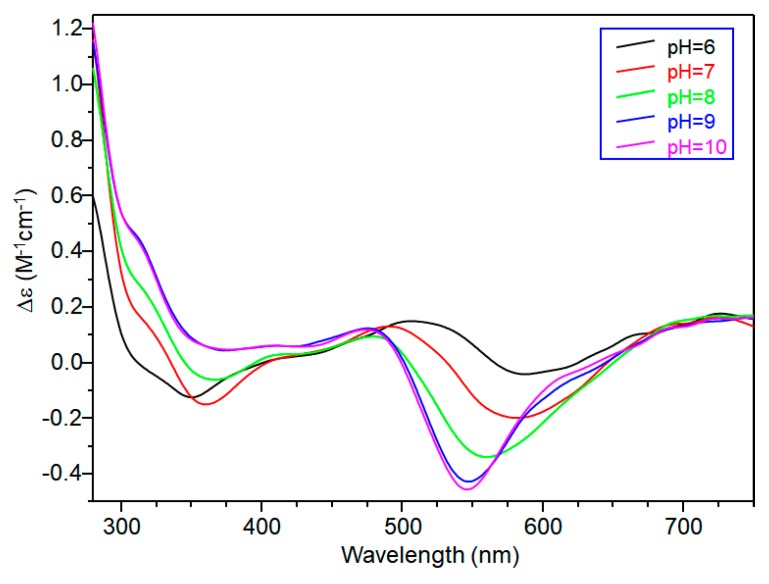
Circular dichroism (CD) spectra of Cu(II)-TrkA(58–64) at different pH values at 1:1 metal-to-ligand molar ratio (L = 1 × 10^−3^ mol·dm^−3^).

CD spectra carried out at pH = 5.8 displayed wide signals at 290 nm and in the 300–360 nm range. However, it was not possible to assign CD signals to the single species [CuL]^2+^. The signal at 350 nm can be assigned to the second N_Imidazole_ → Cu^2+^ charge transfer band, whereas the wide signal around 300 nm was indicative of N^−^_amide_ → Cu^2+^ charge transfer, attributable to the contemporary formation of deprotonated species [CuLH_−1_]^+^ (Figure 3).

Indeed, the p*K* value of 5.97 (Table 1) reveals that macrochelate formation did not hinder the first amide nitrogen deprotonation step, different than observed in peptides having two distal histidine residues [38,39].

**Scheme 2:**
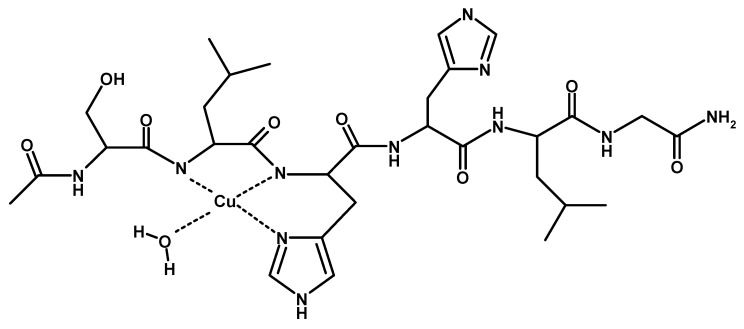
Schematic view of [CuLH_−1_] complex species formed by Cu^2+^ and TrkA(58–64).

[CuLH_−1_]^+^ is the predominant species at pH 7, and the deprotonation p*K* value indicates the formation of six-membered chelate ring, so the nitrogen amide involved belongs to the second histidine residue (Scheme 2) [40,41]. The λ_max_ value in the UV-vis spectra confirmed the formation of a CuN_3_O_1_ chromophore in which the metal ion is bound to two imidazole and one deprotonated amide nitrogen atoms (2N_im_, 1N^−^, O), with the oxygen owing to a carbonyl or a water molecule [35,42]. Accordingly, the band assigned to this species is wide (Figure 2), so the presence of isomers cannot be ruled out. The amide nitrogen deprotonation may occur to the first as well as the second histidine residue. The enlargement of the signal was also observed in the EPR spectra and the magnetic parameters determined from spectra carried out at pH = 6.8 have some uncertainty. However, the low g_||_ value (2.210) and the large hyperfine coupling constant (A_||_ = 0.0194 cm^−1^) are in line with potentiometric and UV-vis data, suggesting the presence of a six-membered chelate ring and a planar disposition of three nitrogen atoms. The CD spectrum of [CuLH_−1_]^+^ at pH 7 clearly shows the increase in both charge transfer bands assigned to imidazole and amide nitrogen atoms and in the d-d transition, confirming the major involvement of the peptide backbone in copper complex formation.

As pH increased, the [CuLH_−2_] species were formed. The p*K* value was indicative of a subsequent deprotonation step of another amide nitrogen. Accordingly, the strong blue shift of 45 nm, observed in the UV-vis spectra related to metal d-d transition, indicated the presence of a fourth nitrogen atom in the metal coordination, resulting in the [2N_Im_, 2N^−^] binding mode [42,43]. The coordination of another amide nitrogen is supported by the increase in dichroic bands in the CD spectrum. EPR parameters agreed with the planar disposition of four nitrogen donor atoms [42,43]. A hypothesis confirmed by the superhyperfine nine-line pattern (2nI + 1; I_N_ = 1) (Figure 2) was observed in the first band of EPR spectra carried out at pH 8.3 (Figure 2).

At higher pH values, [CuLH_−3_]^−^ formed. The spectral parameters were similar to those observed for [CuLH_−2_], so the last deprotonation step can be assigned to a water molecule with the involvement of a hydroxyl group in metal coordination environment. considering that no evident variations were observed in the EPR parameters, the hydroxyl group was not bound in a planar equatorial position [44,45].

**Figure 4:**
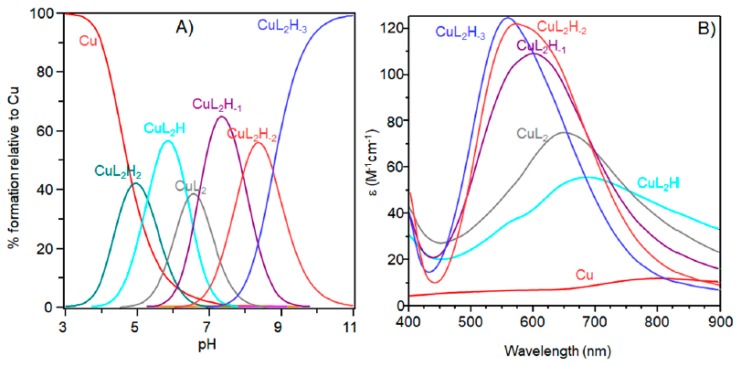
(**A**) Species distribution diagram for Cu-Ac-SLHHLPG-NH_2_ complexes at 1:2 copper-to-ligand molar ratio; and (**B**) UV-vis spectra calculated using the computational program HYPERQUAD [L]= 2 × 10^−3^ M (for clarity, the charges on the speciation plots were omitted).

The stability constants at a 1:2 metal-to-ligand molar ratio are reported in Table 3 and the corresponding diagram distribution obtained is provided in Figure 4.

In this experimental condition, mono-complexes did not form at strong acidic pH. The first complex detected at low pH was [CuL_2_H_2_]^4+^. Spectroscopic parameters (Table 4) were similar to those obtained for [CuL] species, suggesting a [2N_Im_, 2O] copper coordination mode with the involvement of two imidazole nitrogen atoms: one for each ligand peptide and at least one carbonylic oxygen.

**Figure 5:**
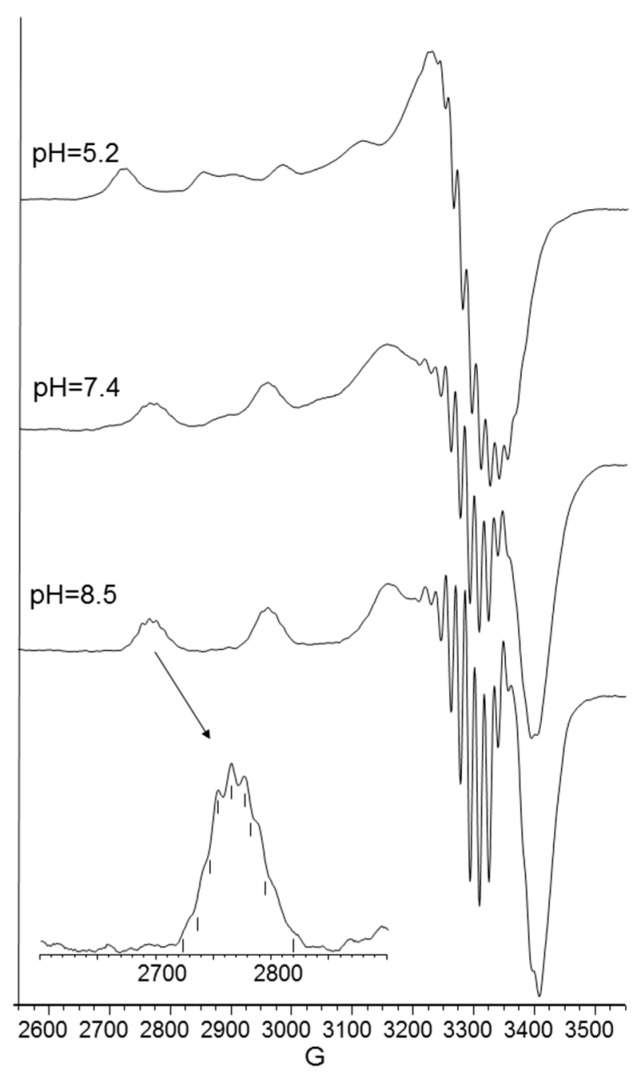
Aqueous frozen solution EPR spectra at 150 K at different pH values of the Cu^2+^-Ac-SLHHLG-NH_2_ system at 1:2 metal-to-ligand molar ratio ([Cu^2+^] = 1 × 10^−3^ mol dm^−3^; [L] = 2 × 10^−3^ mol·dm^−3^). The magnification of the first band of EPR spectrum at pH 8.3 (black arrow) shows superhyperfine pattern superimposed on the first copper feature.

The next bis-complex species formed were [CuL_2_H]^3+^ and [CuL_2_]^2+^. The p*K* values indicated the subsequent deprotonation of an imidazole nitrogen atom. The UV-vis maximum assigned to [CuL_2_H]^3+^ suggested the presence of three imidazole nitrogen in metal coordination, but the signal was large, underlining the presence of isomers and coherent EPR parameters could not be obtained for all species (Table 4). It was possible to determine g_║║_ and A_║║_ values for [CuL_2_H_2_]^4+^ at pH 5.2 (Figure 5) that were similar to those obtained [CuL]^2+^, suggesting a similar Cu(2N_Im_, O_carbonyl_, O_water_) coordination mode. The formation of a bis-complex species suggested that each peptide unit provided one imidazole nitrogen to bind metal ions.

**Figure 6:**
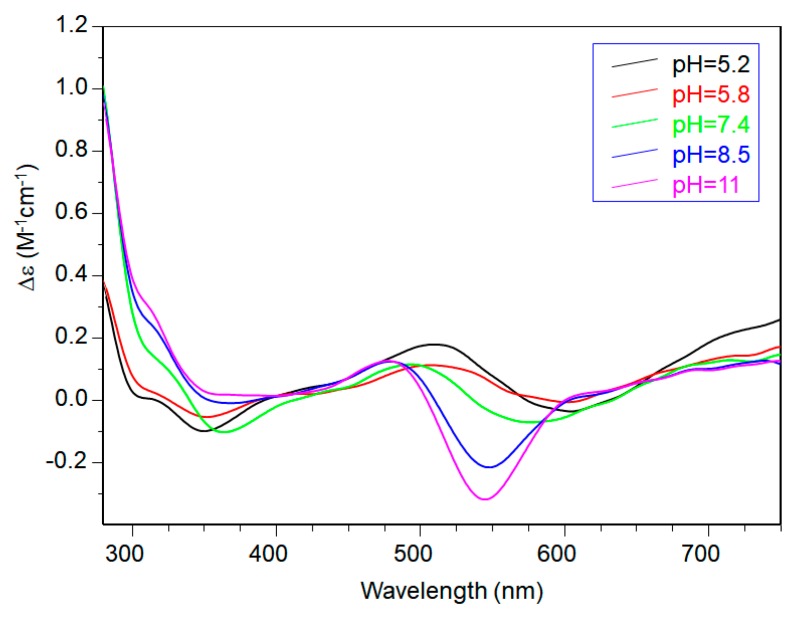
CD spectra of Cu(II)-Ac-SLHHLPG-NH_2_ at different pH values at 1:2 metal-to-ligand molar ratio (L = 2 × 10^−3^ mol·dm^−3^).

CD spectra in the 5–6 pH range show charge transfer band due to amide nitrogen, so an isomer in which one amide is deprotonated and one imidazole is still protonated may be present in both [CuL_2_H]^3+^ and [CuL_2_]^2+^, explaining the difficulty in assigning spectroscopic parameters in this pH range (Figure 6).

At physiological pH, the predominant species was [CuL_2_H_−1_]^+^. The UV-vis λ_max_ was similar to that of [CuLH_−1_]^+^ species but the band was narrow, suggesting fewer isomers were present compared to mono complexes analogous. Notably, the EPR parameters were different and indicative of a [3N_Im_, N^−^] coordination environment [46,47]. The CD spectrum carried out at this pH value was similar to that obtained at a 1:1 metal-to-ligand molar ratio. This indicated that the metal ions experienced a similar chiral environment, so the copper(II) ion was tightly bound to two imidazole and one amide nitrogen of one peptide unit, whereas the other provided one imidazole nitrogen atom [2N_Im_, N^−^ + N_Im_]. The next species bis-complex formed was [CuL_2_H_−2_]. UV-vis, EPR, and CD parameters were similar to those observed for the mono-complex [CuLH_−2_] suggesting a similar [2N_Im_, 2N^−^] coordination environment, with one imidazole and two amide nitrogen of one peptide unit, and the other imidazole was due to a second peptide fragment [1N_Im_, 2N^−^ + N_Im_]. In particular, the EPR spectrum at pH 8.5 showed the superhyperfine nine line pattern of the first band, evidence of the involvement of four nitrogen atoms in the equatorial coordination plane of the metal ion. 

When increasing the pH, [CuL_2_H_−3_] species formed but no changes in the spectroscopic parameters were detected in alignment with that observed at the 1:1 molar ratio. So, at this metal-to-peptide molar ratio, the deprotonation could involve a water molecule.

The potentiometric and spectroscopic data obtained at the 1:2 metal-to-peptide molar ratio indicated that in all pH ranges investigated, one molecule peptide acted as the anchoring site for copper, while the other peptide unit contributed to the metal coordination environment with an imidazole ring to complete tetragonal ligand field. Notably, different from what has been observed in many multi-histidine peptides, there was no evidence for Ac-SLHHLG-NH_2_ species with four imidazoles around the metal ion [48].

## 3. Discussion

The peptide Ac-SLHHLG-NH_2_ encompasses the sequence 58–64 of TrkA, the specific cellular receptor of the NGF neurotrophin. The TrkA receptor has a “pincer” shape conformation, where the domain 58–64 of one unit is close to that of another TrkA unit, forming a pocket where the metal ions could bind [27]. The peptide has two vicinal histidine residues. Potentiometry and spectroscopy studies were carried out at different copper-to-ligand molar ratios to reveal the copper complexes coordination features. At physiological pH and a 1:1 molar ratio, the predominant species was [CuLH_−1_]^+^ in which the metal ions were bound to two imidazole and one deprotonated amide nitrogen atoms. The potentiometric and spectroscopic data obtained at a 1:2 metal-to-peptide molar ratio indicated that in all pH ranges investigated, one molecule peptide acted as the anchoring site for copper, while the other peptide unit contributed to the metal coordination environment with an imidazole ring to complete tetragonal ligand field. At physiological pH, the bis-complex [CuL_2_H_−1_]^+^ was the main species and the copper coordination environment involved three imidazole and one deprotonated amide nitrogen atoms. Notably, different from the reported observations in many multi-histidine peptides, there was no evidence for a Ac-SLHHLG-NH_2_ species with four imidazole around the metal ion [48].

The peptide SSSHPIFHRGEFSV-NH_2_, NGF(1–14) encompasses the first 14 residues of NGF protein and binds one copper ion, which influences its biological activity [20,21]. One of the issues raised about the effect of metal on NGF is the eventual interaction of copper with TrkA receptor.

**Figure 7:**
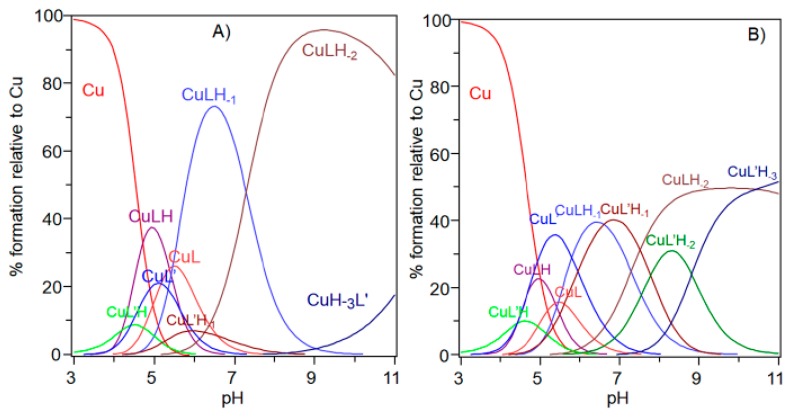
Species distribution diagram for the copper(II) complexes formed with the NGF(1–14) (L) and Ac-SLHHLPG-NH_2_ (L’) at (**A**) 1:1:1 Cu,L,L’ molar ratio and (**B**) 1:1:2 Cu,L,L’ ([Cu^2+^] = 1 × 10^−3^ mol dm^−3^).

The comparison of the potentiometric results obtained for copper complexes formed with Ac-SLHHLG-NH_2_ and NGF(1–14) are reported in Figure 7. At a 1:1 metal-to-ligand molar ratio, NGF(1–14) was a stronger ligand for copper. The receptor has a dimeric structure and comparison with two equivalent of Ac-SLHHLG-NH_2_ showed that copper ions were distributed between two systems.

These data, even if obtained on small model systems, may suggest a potential competition for copper binding between NGF and TrkA, depending on the amount of metal ions in the synaptic space. The NGF recognition processes, with their specific cellular receptors, may be influenced by copper ions suggesting a starting point for future research on NGF activity.

## 4. Materials and Methods

### 4.1. Materials

*N*-Fluorenylmethoxycarbonyl (Fmoc)-protected amino-acids, and NovaSyn-TGR resin (loading 0.18 mM/g, 0.33 mM scale synthesis)” resin were purchased by Merck (Darmstadt, Germany); *N*,*N*-diisopropyl-ethylamine (DIEA), *N*,*N*-dimethylformamide (DMF, peptide synthesis grade), piperidine, dimethylformammide (DMF), *N*-hydroxybenzotriazole (HOBt), 2-(1-H-benzotriazole-1-yl)-1,1,3,3-tetramethyluronium tetrafluoroborate (TBTU), triisopropylsilane (TIS), and trifluoroacetic acid (TFA), were purchased from Sigma-Aldrich (Munich, Germany). Peptide solution was prepared dissolving lyophilized peptide in water. Ultrapure Milli-Q water (resistivity > 18 MΩ·cm^−1^) was used for all experiments.

### 4.2. Peptide Synthesis and Purification

The peptide Ac-SLHHLPG-NH_2_ was synthesized with standard Fmoc chemistry through solid-phase peptide synthesis on a Biotage initiator^+^ Alstra™ microwave peptide synthesizer (Biotage, Uppsala, Sweden). The synthesis was performed on TGR resin (0.25 mM·g^−1^) on a 0.11 mM scale using a 30 mL reactor vial. The coupling reactions were carried out by using 5-fold excess of amino acid, 5 equivalent of HOBt/TBTU/DIPEA in DMF, under mixing for 10 min at room temperature. Fmoc deprotection steps were performed at room temperature using 20% of piperidine in DMF for 15 min. The N-terminal amino group was acetylated using a DMF solution containing acetic anhydride (6% *v*/*v*) and DIEA (5% *v*/*v*). The resin was washed with dichloromethane and dried on a synthesizer. Other experimental details have been reported previously [27].

The peptide was purified by preparative reverse-phase chromatography (rp)-HPLC using a Varian PrepStar 200 (model SD-1) equipped with a Prostar photodiode array detector (Varian, Palo Alto (CA), USA). The protocol used to purify peptide was: from 0 to 5 min isocratic gradient in 0% B; linear gradient from 0 to 10% B over 15 min; and isocratic gradient in 10% B from 15 to 30 min [27]. Peptide was characterized by means of electrospray ionization mass spectrometry (ESI-MS) (Thermo Fisher Scientific instruments, Waltham, MA, USA). Ac-SLHHLPG-NH_2_: [R_t_ = 23.1 min]. Calculated mass for C_36_H_56_N_12_O_9_ M = ESI-MS [Obsd. *m/z*: (M + H)^+^ 801.8].

### 4.3. Potentiometric Titrations

Potentiometric titrations were carried out by using a Titrando 905 automatic titrator (Metrhom, Herisau, Switzerland) and a combined glass-Ag/AgCl electrode (Metrohm). Under an argon atmosphere, the titrated solutions (2.0 mL) were thermostatted at 298 K. A calibration of the electrode was carried out before any experiment by titrating a 4 mM solution of HNO_3_ (0.1 M in KNO_3_) with a 0.1 M KOH solution free of CO_2_ between pH 2.4 and 3.3.

KOH solution (0.1 M) was also used to titrate either solutions of the peptide to determine protonation constants or the peptides in the presence of Cu^2+^ to obtain complexation constants. The concentration of the peptide was 1 × 10^−3^ M for protonation titrations and ranged from 1 to 2 × 10^−3^ M for the complexing titrations. Both protonation and complexation independent titrations were performed four times. The initial pH value was adjusted to 2.4 by adding HNO_3_ 0.2 M. The final pH was always 11. Metal ion/ligand ratios between 1:2 to 1:1 were employed. Systematic errors and reproducibility were avoided determining the EMF values of each experiment at different time intervals.

All experimental data were analysed by using the HYPERQUAD 2003 program [49]. It minimizes the square error sum of the measured electrode potentials using a non-linear iterative refinement of the sum of the squared residuals, *U*, and also allows for the simultaneous refinement of data from different titrations:

(1) $$U=\sum {\left(E_{\exp}-E_{\mathrm{calc}}\right)}^{2}$$

where *E*_exp_ and *E*_calc_ represent experimental and calculated electrode potentials, respectively. Stability constant values errors are reported as three times standard deviations (3σ). Equation (2) shows the formation reaction equilibria of ligands with protons and copper(II):

(2) $$\mathrm{pCu}+\mathrm{qL}+\mathrm{rH}⇆{\mathrm{Cu}}_{p}L_{q}H_{r}$$

where L is the peptide Ac-SLHHLPG-NH_2_.

The stability constant βpqr is defined in Equation (3):

(3) $$\beta \mathrm{pqr}=\left[{\mathrm{Cu}}_{p}L_{q}H_{r}\right]/{\left[\mathrm{Cu}\right]}^{p}{\left[L\right]}^{q}{\left[H\right]}^{r}$$

Finally, species distribution diagrams as a function of pH were determined using the Hyss program [50].

### 4.4. Spectroscoscopic Studies

#### 4.4.1. UV-Vis Measurements

UV-vis spectra were carried out on an Agilent 8453 spectrophotometer (Agilent Technologies, Santa Clara, CA, USA). at room temperature. The peptide and copper(II) concentrations were the same used in the potentiometric titrations. The spectra were carried out in the 200–800 nm range. Combined spectroscopic and potentiometric metal-complex titrations were performed in a 3 mL quartz cuvette with a 1 cm path length. The experiment was replicated three times. The spectroscopic data were processed by the HYPERQUAD program [49].

#### 4.4.2. CD Measurements

CD spectra were obtained at 25 °C in a constant nitrogen flow on a Jasco model 810 spectropolarimeter (Jasco, Easton, MD, USA). The spectra were recorded in the 275–750 nm range, as an average of 3 scans (scan rate 50 nm·min^−1^; resolution 0.1 nm; path length 1 cm). The peptide and copper(II) concentration were identical to those used in the potentiometric measurements. The pH of aqueous solution was adjusted by adding NaOH.

#### 4.4.3. EPR Measurements

X-band EPR spectra were obtained on a continuous wave performed on a Bruker Elexsys E500 CW-EPR spectrometer (Bruker, Billerica, MA, USA) equipped with a Super-X microwave bridge, operating at 9.3–9.5 GHz, and a Bruker Super High QE (SHQE) cavity resonator. The *g* factor was calibrated in the experimental conditions using a Bruker strong pitch (*g* = 2.0028). The spectra were recorded at 150 K with a variable temperature apparatus (ER4131VT). The *g* and *A* values were obtained directly from the experimental EPR spectra, calculating them from the 2nd and 3rd line to avoid second order effects [51]. The spectra were recorded as an average of 6 scans, microwave frequency 9.344–9.376 GHz, modulation frequency 100 kHz, modulation amplitude 0.2–0.6 mT, time constant 164–327 ms, sweep time 2.8 min, microwave power 20–40 mW, and receiver gain 50–60 dB. The Cu^2+^ peptide solutions were prepared at 1:1 and 1:2 metal-to-ligand ratios in the concentration range of 1 to 2 mM. The copper used in EPR measurements was isotopically pure ^63^Cu, taken from a 0.05 M ^63^Cu(NO_3_)_2_ stock solution. The pH of aqueous solution was adjusted by adding NaOH. 10% of methanol was added to aqueous solutions to enhance spectral resolution at low temperatures.

## Figures and Tables

**Table 1:** Stability constants logβ(pqr) and p*K* values of the complex species formed by Cu^2+^ with Ac-SLHHLG-NH_2_ at 1:1 metal-to-ligand molar ratio. (T = 298 K; I = 0.1 M KNO_3_) *.

Species (pqr)	logβ	p*K* (n/m)
CuLH	10.61 (9)	-
CuL	6.19 (3)	4.42
CuLH_−1_	0.23 (5)	5.97
CuLH_−2_	−7.55 (4)	7.78
CuLH_−3_	−16.36 (6)	8.80

* Standard deviations (three values) are given in parentheses. Charges are omitted for clarity.

**Table 2:** Spectroscopic parameters of copper(II) complexes with Ac-SLHHLG-NH_2_ peptide. Metal-to-ligand molar ratio 1:1, [L] = 1 × 10^−3^ M.

pH	Species	UV-Vis		CD	EPR	
		λ (nm) (ε (M^−1^·cm^−1^))		λ (nm) (Δε (M^−1^·cm^−1^))	g_║║_	A_║║_ (10^−4^ cm^−1^)
5.8	CuL	650	(50)	280 (0.65), 350 (−0.30), 511 (0.30), 723 (0.25)	2.315 (2)	145 (3)
6.8	CuLH_−1_	595	(95)	280 (1.40), 362 (−0.22), 482 (0.17), 585 (−0.71), 723 (0.30)	2.210 (3)	194 (5)
8.3	CuLH_−2_	550	(105)	280 (1.52), 362 (−0.10), 474 (0.24), 549 (−0.80), 717 (0.19)	2.206 (1)	202 (2)
9–11	CuLH_−3_	550	(95)	280 (1.90), 477 (0.23), 547 (−0.78)	2.206 (1)	202 (2)

**Table 3:** Stability constants logβ(pqr) and p*K* values of the complex species formed by Cu(II) with Ac-SLHHLG-NH_2_ at 1:2 metal-to-ligand molar ratio. (T = 298 K; I = 0.1 M KNO_3_) *.

Species (pqr)	logβ	p*K* (*n*/*m*)
CuL_2_H_2_	22.12 (5)	-
CuL_2_H	16.85 (3)	5.27
CuL_2_	10.46 (5)	6.39
CuL_2_H_−1_	3.77 (4)	6.69
CuL_2_H_−2_	−4.17 (4)	7.94

* Standard deviations (three values) are provided in parentheses. Charges are omitted for clarity. [L] = 2 × 10^−3^ M.

**Table 4:** Spectroscopic parameters of copper(II) complexes with Ac-SLHHLG-NH_2_ peptide at metal-to-ligand molar ratio 1:2 ([L] = 2 × 10^−3^ M).

pH	Species	UV-Vis		CD	EPR	
		λ (nm) (ε (M^−1^·cm^−1^))		λ (nm) (Δε (M^−1^·cm^−1^))	g_║_	A_║_ (10^−4^ cm^−1^)
5.2	CuL_2_H_2_	675	(55)	−	2.310 (2)	142 (3)
5.8	CuL_2_H	650	(75)	280 (0.44), 349 (0.06), 516 (0.13), 600 (−0.03), 725 (0.13)	−	−
7.4	CuL_2_H_−1_	598	(110)	280 (1.16), 360 (−0.11), 491 (0.14), 568 (−0.09), 723 (0.12)	2.249 (2)	192 (3)
8.5	CuL_2_H_−2_	555	(120)	280 (1.20), 474 (0.15), 549 (−0.23), 723 (0.12)	2.206 (1)	202 (2)
11	CuL_2_H_−3_	550	(125)	280 (1.05), 481 (0.15), 547 (−0.35)	2.206 (1)	202 (2)
